# Reduction in Biogenic Amine Content in *Baechu* (Napa Cabbage) Kimchi by Biogenic Amine-Degrading Lactic Acid Bacteria

**DOI:** 10.3390/microorganisms9122570

**Published:** 2021-12-13

**Authors:** Junsu Lee, Young Hun Jin, Alixander Mattay Pawluk, Jae-Hyung Mah

**Affiliations:** Department of Food and Biotechnology, Korea University, 2511 Sejong-ro, Sejong 30019, Korea; jpang@korea.ac.kr (J.L.); younghoonjin3090@korea.ac.kr (Y.H.J.); alixei@korea.ac.kr (A.M.P.)

**Keywords:** biogenic amine, lactic acid bacteria, biogenic amine degradation, multicopper oxidase, *Baechu* kimchi, fermentation

## Abstract

This study was performed to mine biogenic amine (BA)-degrading lactic acid bacteria (LAB) from kimchi and to investigate the effects of the LAB strains on BA reduction in *Baechu* kimchi fermentation. Among 1448 LAB strains isolated from various kimchi varieties, five strains capable of considerably degrading histamine and/or tyramine were selected through in vitro tests and identified as *Levilactobacillus brevis* PK08, *Lactiplantibacillus pentosus* PK05, *Leuconostoc mesenteroides* YM20, *L. plantarum* KD15, and *Latilactobacillus sakei* YM21. The selected strains were used to ferment five groups of *Baechu* kimchi, respectively. The LB group inoculated with *L. brevis* PK08 showed the highest reduction in tyramine content, 66.65% and 81.89%, compared to the control group and the positive control group, respectively. Other BA content was also considerably reduced, by 3.76–89.26% (five BAs) and 7.87–23.27% (four BAs), compared to the two control groups, respectively. The other inoculated groups showed similar or less BA reduction than the LB group. Meanwhile, a multicopper oxidase gene was detected in *L. brevis* PK08 when pursuing the BA degradation mechanism. Consequently, *L. brevis* PK08 could be applied to kimchi fermentation as a starter or protective culture to improve the BA-related safety of kimchi where prolific tyramine-producing LAB strains are present.

## 1. Introduction

Biogenic amines (BAs), such as tyramine, histamine, putrescine, cadaverine, spermidine, spermine, tryptamine, and β-phenylethylamine, are basic nitrogenous organic compounds that are produced mainly though microbial decarboxylation of precursor amino acids [1]. Although the BAs are generally vital for biological functions such as the regulation of the nervous system, the cardiovascular system, and body temperature, the excessive consumption of foods with high levels of BAs, as well as inhibition and deficiency of amine oxidase by specific inhibitors, occasionally induces several adverse health effects [2]. Among BAs, tyramine and histamine (toxic BAs) are considered to be the most toxic due to numerous toxicological effects [2,3]. The over-consumption of foods containing high levels of these BAs may potentially cause histamine poisoning (e.g., scombroid poisoning) or tyramine toxicity (e.g., cheese crisis) with various symptoms including headache, nausea, respiratory difficulties, migraine, vomiting, brain haemorrhage, heart failure, hypo- or hypertension, etc. [4,5]. Additionally, polyamines, including cadaverine, putrescine, spermine, and spermidine, have the potential to interrupt the metabolization of tyramine and histamine, in turn, enhancing their toxicity, and may possibly cause the formation of carcinogenic N-nitrosamines by reacting with nitrite [6,7]. According to the EFSA [3], an intake of 50 mg histamine or 600 mg tyramine per meal showed no adverse effects for healthy individuals, while these concentrations were much lower for individuals taking monoamine oxidase inhibitors. Several studies have suggested <100 mg/kg of histamine, <100 to 800 mg/kg of tyramine, <30 mg/kg of β-phenylethylamine, and <1000 mg/kg of total BAs as the safety criteria (upper limits) for BAs in food [1,8]. Although toxicity limits of BAs have been studied [9,10,11,12,13], there are no regulations, except for histamine, set by governments and international organizations. Even more limiting, current regulations by the European Commission (EC) and the U.S. Food and Drug Administration (FDA) are in place only in regard to the histamine content of 50 to 100 mg/kg in fish and 400 mg/kg in fish sauces and other fish products [14,15].

The term kimchi describes a wide variety of traditional Korean lactic acid-fermented vegetable products that are commonly produced through natural fermentation without the use of a starter culture. *Baechu* kimchi (napa cabbage kimchi), in particular, is prepared by mixing salted napa cabbage with seasoning ingredients composed of radish, garlic, ginger, green onion, and red pepper powder, followed by fermentation at low temperatures (typically at 2 to 10 °C) for a few weeks [16,17]. Other seasoning ingredients such as *Jeotgal* and *Aekjeot* (salted and fermented seafood products) are also frequently added [18]. It is known that the microflora of kimchi is composed mainly of lactic acid bacteria (LAB) species, including *Lactobacillus*, *Leuconostoc*, and *Weissella* spp., which are the major microorganisms responsible for kimchi fermentation [19,20]. The microflora of kimchi varieties differs considerably depending on the main and seasoning ingredients used and the fermentation conditions, as well as physicochemical and biological factors in preparing the kimchi [16]. In the processes of kimchi fermentation, diverse LAB derived from the raw ingredients play a vital role in producing vitamins, organic acids, carbon dioxide, ethanol, bacteriocins, and prebiotic factors, which affect the organoleptic properties of the final kimchi product [21,22]. Several studies have reported on the numerous beneficial properties and functionalities of kimchi, including anti-mutagenic [23], anti-diabetic [24], anti-obese [25], anti-aging [26], antioxidative [27], and anti-carcinogenic effects [28]. Despite the numerous benefits of kimchi, potentially hazardous substances, such as BAs, may be formed by microbial enzymatic activity and/or by undesirable influences of the ingredients used in kimchi preparation [29,30]. There are numerous reports [31,32,33,34,35] on tyramine and/or histamine concentrations in different types of kimchi, including *Beachu* kimchi, that were higher than the upper limits suggested by Ten Brink et al. [8]. For instance, several studies have described commercial *Baechu* kimchi as containing histamine up to 142.3 mg/kg and tyramine up to 118.2 mg/kg [31,33]. Surprisingly, another previous study reported commercial *Baechu* kimchi to contain histamine up to 5350 mg/kg [35]. Histamine contents of up to 386.03 mg/kg and tyramine contents of up to 181.10 mg/kg in other types of kimchi, including *Kkakdugi* (kimchi made mainly of diced radish), *Chonggak* kimchi (kimchi made mainly of ponytail radish), *Pa* kimchi (kimchi made mainly of green onion), and *Gat* kimchi (kimchi made mainly of mustard leaf), have also been reported in previous studies [32,34].

The majority of solutions for reducing BAs in foods have been well reviewed in literature, and include additives, irradiation, packaging, antimicrobial compounds, starter cultures, and control or adjustment of environmental factors [36,37]. Particularly, in fermented foods, utilizing microorganisms capable of degrading and/or incapable of producing BAs as starter cultures is considered to be one of the most promising strategies, because they provide less or no unfavorable organoleptic and unhealthy alterations and insignificant changes in microbial communities in the foods compared to other treatments [30,37]. Diverse LAB species that have been studied to degrade BAs in fermented foods include: *Companilactobacillus farciminis* (formerly *Lactobacillus farciminis*), *Lactiplantibacillus plantarum* (formerly *Lactobacillus plantarum*)*,* and *Pediococcus acidilactici* for histamine, tyramine, and putrescine reduction in wine [38]; *Latilactobacillus sakei* (formerly *Lactobacillus sakei*) for histamine reduction in fish slurry [39]; *L. plantarum* for tyramine, putrescine, cadaverine, histamine, and spermidine reduction in sausage [40]; *Lacticaseibacillus paracasei* (formerly *Lactobacillus paracasei*) for histamine and tyramine reduction in cheese [41]; and *Latilactobacillus curvatus* (formerly *Lactobacillus curvatus*) for total BA reduction in fermented meat [42]. In addition, it was confirmed that the multicopper oxidase gene (MCO) found in LAB strains may be primarily responsible for BA degradation [38,41,42].

Although the capability of LAB strains to degrade BAs in fermented foods has been reported in previous studies regarding kimchi products, few reports are available on the BA degradation effects of BA-degrading LAB strains. Therefore, this study was performed to seek BA-degrading LAB that originated from kimchi and to pursue a mechanism of BA degradation using a primer set that detects the MCO gene. The practical effects of the selected BA-degrading LAB strains on BA reduction during fermentation of *Baechu* kimchi were also investigated. This is the first study on the use of BA-degrading LAB strains originating from kimchi, which carry the MCO gene, as starter (or protective) cultures to reduce the content of BAs, including polyamines and toxic BAs, in kimchi.

## 2. Materials and Methods

### 2.1. Bacterial Strains Used

A total of 1448 LAB strains were isolated from six types of kimchi varieties, including *Baechu* kimchi (napa cabbage kimchi), *Kkakdugi* (kimchi made mainly of diced radish), *Chonggak* kimchi (kimchi made mainly of ponytail radish), *Yeolmu* kimchi (kimchi made mainly of young radish), *Gat* kimchi (kimchi made mainly of mustard leaf), and *Pa* kimchi (kimchi made mainly of green onion), including those from previous studies (532 strains) [32,34] and in the present study (916 strains). The reference LAB strains used in this study, including *Levilactobacillus brevis* JCM 1170, *L. helveticus* KCCM 40989, *L. plantarum* KCTC 3108, *L. sakei* KCCM 43213, *L. casei* KCCM 12452, *L. paracasei* KCTC 3510, *Limosilactobacillus fermentum* KCTC 3112, *Lentilactobacillus buchneri* KCTC 5064, *Leu. mesenteroides* KCTC 3505, and *Leu. citreum* KCCM 12030, were purchased from the Korean Culture Center of Microorganisms (KCCM; Seoul, South Korea), the Japan Collection of Microorganisms (JCM; Saitama, Japan), and the Korean Collection for Type Cultures (KCTC; Daejeon, South Korea). All strains were cultured in de Man, Rogosa, and Sharpe (MRS; Laboratorios Conda, Madrid, Spain) broth at 37 °C for 48 h and kept in glycerol (20%, *v*/*v*) in a deep freezer at −70 °C.

### 2.2. Determination of Histamine and Tyramine Degradation by LAB Strains

Degradation of toxic BAs by LAB strains was assessed following the method developed by Leuschner et al. [43], with slight modifications. To prepare resting cells, 10 µL of glycerol stock of each LAB strain were inoculated in 5 mL of MRS broth. After aerobic incubation at 37 °C for 48 h, a loopful of the cultured broth was streaked on MRS agar, which was incubated under the same conditions. A single colony was inoculated in 5 mL of MRS broth. After aerobic incubation at 37 °C for 48 h, 200 µL of the cultured broth was transferred into 10 mL of MRS broth. After aerobic incubation at 37 °C for 48 h under shaking at 200 rpm, the cultured broth was centrifuged at 9000× *g* for 10 min at 4 °C. The pellet was washed twice with sodium phosphate buffer (0.05 M, pH 7.00; 4.0962 g of Na_2_HPO_4_ and 2.537 g of NaH_2_PO_4_ dissolved in 1 L of distilled water, all from Sigma–Aldrich Chemical Co., St. Louis, MO, USA). The cell pellet was resuspended in 10 mL of the same buffer but that contained 0.5 mM histamine and 0.5 mM tyramine (all from Sigma–Aldrich, *w*/*v*). The cell suspension was reacted with shaking at 200 rpm for 24 h at 30 °C, an optimal temperature for amine oxidase activity. The cell suspension was then filtered through a 0.2 µm membrane filter (Millipore Co., Bedford, MA, USA) using a syringe and immediately analyzed by HPLC for toxic BA degradation.

To accelerate the screening of toxic BA-degrading LAB strains, an alternative method to measure toxic BA degradation by LAB strains in MRS broth was used [39]. Briefly, after activation of the LAB strains in MRS broth under the same incubation conditions above, 200 µL of the cultured broth were inoculated into 10 mL of MRS broth supplemented with 50 ppm (*w*/*v*) of histamine or tyramine. The MRS broth containing histamine or tyramine without bacterial cells was used as a control. All broths were incubated statically at 30 °C for 24 h. Subsequently, the broth culture was filtered through a 0.2 µm membrane and one milliliter of the filtered broth culture was immediately analyzed using HPLC. After assessing the degradation activity of LAB strains using this method, selected LAB strains showing higher degradation activity were retested for degradation activity using the method of measurement in buffer as described above.

### 2.3. Identification of LAB Strains

The LAB strains selected based on toxic BA degradation were identified through 16S rRNA gene sequencing with universal bacterial primers 518F and 805R, as in the previous studies [32,34].

### 2.4. Baechu Kimchi Fermentation with the Selected LAB Strains Capable of Degrading Toxic BAs

To investigate the effects on the reduction in tyramine, histamine, and other BAs by selected LAB strains, *Baechu* kimchi was prepared using the recipe described by Kim et al. [44]. Napa cabbages were obtained from a grocery store in Sejong, South Korea. Napa cabbages were quartered and soaked in 10% salt brine (*w*/*v*) for 10 h. Then, salted napa cabbages were washed thrice with tap water. After draining for 3 h at room temperature, 100.0 g of salted napa cabbage were mixed with 13.0 g julienned white radish, 3.5 g red pepper powder, 2.2 g *Myeolchi*-*aekjeot* (Korean salted and fermented anchovy sauce), 2.0 g green onion, 1.4 g garlic, 1.0 g sugar, and 0.6 g ginger. The salinity of the kimchi was adjusted to 2.5% by adding deionized water.

The *Baechu* kimchi was divided into seven experimental groups—one non-inoculated group (C group) and six inoculated groups. The inoculated groups were LB, LT, LM, LP, and LS groups inoculated with each of the five selected LAB strains (*L. brevis* PK08, *L. pentosus* PK05, *Leu. mesenteroides* YM20, *L. plantarum* KD15, and *L. sakei* YM21, respectively) capable of degrading toxic BAs and incapable of producing both toxic BAs. The C group fermented naturally without inoculum and the PC group inoculated with *L. brevis* PK11, capable of largely producing tyramine (295.63 ± 12.44 µg/mL), served as the control and positive control, respectively. Each LAB inoculum was inoculated into *Baechu* kimchi (except for the C group) at a level of about 7 Log CFU/g. All the experimental groups were placed in plastic containers (250 × 150 × 200 mm^3^) and fermented for three days at 25 °C, a temperature commonly used for kimchi fermentation experiments [34]. All kimchi samples belonging to the seven experimental groups were prepared in duplicate.

### 2.5. Measurements of Physicochemical and Microbial Properties

For all measurements of physicochemical and microbial properties, *Baechu* kimchi broth was sampled following previous studies [32,34,45]. The physicochemical properties, including pH, acidity, salinity, and water activity, and the microbial properties, including the counts of total viable mesophilic bacteria and LAB, of the samples from respective experiment groups were measured as in the previous studies [32,34,45]. Briefly, the pH and water activity (a_w_) of *Baechu* kimchi samples were determined using a pH meter (Orion 3-star Benchtop pH meter; Thermo Scientific, Waltham, MA, USA) and a water activity meter (AquaLab Pre; Meter Group, Inc., Pullman, WA, USA), respectively. The acidity and salinity of *Baechu* kimchi samples were determined according to the AOAC methods, respectively [46]. The counts of total viable mesophilic bacteria and LAB were determined on Plate Count Agar (PCA; Becton Dickinson, Sparks, MD, USA) and MRS agar, respectively. 

### 2.6. Analyses of BAs in Baechu Kimchi Samples and Assay Menstrua

BA analyses in *Baechu* kimchi samples and assay menstrua were conducted according to protocols described in a previous study [34]. For an internal standard, 1,7-Diaminoheptane (1 mg/mL; Sigma) was used. Tyramine hydrochloride, histamine dihydrochloride, cadaverine dihydrochloride, putrescine dihydrochloride, spermine tetrahydrochloride, spermidine trihydrochloride, tryptamine, and β-phenylethylamine hydrochloride were used to prepare standard solutions (all from Sigma). The analysis procedures included extraction of BAs, preparation of BA standard solutions, derivatization of BAs, and chromatographic separation of BAs. Both limits of detection and limits of quantification for all BAs were about 0.10 µg/mL in standard solutions and 0.01–0.10 mg/kg and 0.02–0.31 mg/kg, respectively, in food matrices [47].

### 2.7. Detection and Identification of Multicopper Oxidase Gene in LAB Strains

Polymerase chain reaction (PCR) was performed to detect a gene encoding a BA-degrading enzyme, MCO, responsible for BA degradation in the LAB strains. To design specific gene primers, the DNA sequence of the MCO gene for *L. brevis* was retrieved from the National Center for Biotechnology Information (NCBI). The primer set, Lbre-F (5′-TGCCCGTTACGTGAGACTAC-3′) and Lbre-R (5′-GACTTGTGCTGAACGTGCTG-3′), was designed for detection of the target gene in the *L. brevis* strains and in the other strains using the NCBI Primer-BLAST [48]. The PCR-amplified DNA fragment was 390 base pairs in length.

Afterwards, genomic DNA extraction from bacterial cultures was conducted using the G-spinTM genomic DNA extraction kit (Intron Biotechnology, Inc., Seongnam, South Korea). The 50-µL PCR reaction mixture consisted of 1.5 µL of 10 pM of each primer, 0.25 µL of Ex Taq DNA polymerase (5.0 units/µL; Takara Biotechnology Co. Ltd., Tokyo, Japan), 5 µL of 10× Ex Taq buffer, 4 µL of dNTPs (each at 0.2 mM; Takara), and 37.75 µL of 200 ng genomic DNA in deionized water. PCR amplification was carried out using a thermal cycler (DNA Engine Peltier T100 thermal cycler, Bio-Rad Laboratories, Hercules, CA, USA). The PCR conditions were as follows: an initial denaturation at 94 °C for 1 min, followed by 30 cycles of denaturation at 94 °C for 30 s, annealing at 54 °C for 40 s and extension at 72 °C for 1 min, and final extension at 72 °C for 5 min. 

The PCR products were electrophoresed on agarose gel (1.2%, *w*/*v*; Duchefa Biochemie, Haarlem, The Netherlands) in 0.5× Tris-borate-EDTA buffer (TBE; Bio Basic, Markham, ON, Canada) with ethidium bromide (0.5 µg/mL; Sigma–Aldrich) by electrophoresis at 100 V for 30 min. The 1 kb DNA Ladder (Elpis Biotech, Daejeon, South Korea) was used as a size marker. Subsequently, the gel was visualized on a transilluminator (SMU-01 Slider UV Imager; Maestrogen Inc., Hsinchu, Taiwan) under ultra-violet light and photographed. 

The PCR products were also sequenced and compared with the MCO gene of *L. brevis*, available in the GenBank database using the Basic Local Alignment Search Tool [49].

### 2.8. Statistical Analyses

All physicochemical measurements were performed in triplicate, while microbial measurements were conducted in duplicate. Fermentation experiments with the seven experimental kimchi groups were conducted independently in duplicate. Data were presented as means and standard deviations of duplicates or triplicates. Error bars in all plots indicate the standard deviations determined from the fermentation experiments in duplicate. The determination of significant differences was conducted using the one-way analysis of variance (ANOVA) with Fisher’s pairwise comparison in Minitab statistical software (Version 17.1.0. Minitab Inc., State College, PA, USA), and differences with a probability (*p*) value of <0.05 were considered statistically significant.

## 3. Results and Discussion

### 3.1. Histamine and Tyramine Degradation Activity of LAB Strains Isolated from Kimchi Products

To screen LAB strains capable of degrading toxic BAs (i.e., histamine and tyramine), a total of 1448 LAB strains, consisting of 532 LAB strains used in previous studies [32,34] and kept in the laboratory, and 916 LAB strains newly obtained in the present study, isolated from six types of kimchi products, including *Baechu* kimchi, *Kkakdugi*, *Chonggak* kimchi, *Yeolmu* kimchi, *Gat* kimchi, and *Pa* kimchi, were tested for toxic BA degradation activity in a sodium phosphate buffer and/or in culture media containing BAs. 

Most of the LAB strains (1373 strains) tested displayed low or no degradation of toxic BAs, showing a degradation rate of less than 10% (Tables S1 and S2). Contrastingly, the other 75 LAB strains showed the capability to degrade more than 10% of either toxic BA, and were subject to further testing, though only in culture media to observe the results quickly, to confirm the reproducibility of their degradation activity. As a result, five LAB strains exhibited reproducibility for a toxic BA degradation rate of 10% or more. Overall, the ranges of the highest degradation rates of toxic BAs by each of the five selected LAB strains were as follows: from 1.39 to 10.81% for histamine and from 7.43 to 14.97% for tyramine in buffer, and from 1.59 to 8.56% for histamine and from 11.68 to 14.88% for tyramine in culture media (Table 1). Through 16S rRNA sequencing, the five selected LAB strains, PK08, PK05, YM20, KD15, and YM21, were identified as *L. brevis* (accession number NR116238), *L. pentosus* (accession number NR029133), *Leu. mesenteroides* (accession number NR118557), *L. plantarum* (accession number NR117813), and *L. sakei* (accession number NR042443), respectively (Table 1). The identified species have often been found in *Baechu* kimchi, and among them, *L. brevis*, *Leu. mesenteroides*, and *L. plantarum* have been especially considered to be representative LAB species associated with kimchi fermentation [22,50,51]. Considering the results described above, these five LAB strains were selected for further use in kimchi fermentation. In addition, all other strains identified as one of *L. brevis*, *L. pentosus*, *Leu. mesenteroides*, *L. plantarum*, and *L. sakei*, like the five selected LAB strains, showed lower degradation of both toxic BAs.

Regarding the assay menstrua, while determination in buffer has been shown to precisely measure degradation rates, determination in culture media can obtain results more quickly [39,43]. Although the former may be considered more reliable, there was no statistically significant difference between the results of almost all the degradation rates obtained from both the buffer and culture media (Table 1). Based on these results, it seems preferable to use the latter to quickly select toxic BA-degrading LAB strains.

Alvarez and Moreno-Arribas [52] reviewed that foodborne bacteria, such as *Lactobacillus* spp., *Pediococcus* spp., *Rhodococcus* spp., *Arthrobacter* spp., *Micrococcus* spp., *Brevibacterium* spp., *Bacillus* spp., *Staphylococcus* spp., and *Oenococcus* spp., could degrade BAs using their amine-oxidation activity. Dapkevicius et al. [39] found that 4 of 48 LAB strains isolated from fish pastes were capable of degrading histamine in assay broth, showing a degradation rate ranging from 19.61 to 56.18%, and were all identified as *L. sakei*. Herrero-Fresno et al. [53] reported that 17 of 157 LAB strains isolated from cheeses degraded histamine and tyramine in the ranges of 12.75 to 47.90% and 14.92 to 56.52%, respectively, in broth culture, and were all identified as *Lacticaseibacillus casei* (formerly *Lactobacillus casei*). The previous studies are in agreement with this study, showing the capability of LAB strains isolated from kimchi products to degrade toxic BAs. In this study, however, the capability of degrading tyramine or histamine by the selected LAB strains appeared to be slightly lower than that of LAB strains reported in the aforementioned studies. The BA content of kimchi may be one of the reasons for the difference in the degradation activity of LAB strains in the current study compared to previous studies. It is known that the BA content in kimchi is low compared to other fermented foods, likely due to the fact that the amount of precursor amino acids of BAs is lower in kimchi than in protein-rich fermented foods such as cheese, fermented seafood products, fermented soybean foods, fermented sausage, etc., which provide abundant precursor amino acids [29,30,31,37]. Therefore, it seems inevitable that the BA degradation activity of LAB strains isolated from kimchi with small amounts of degradable BAs would be relatively low. To isolate LAB strains possessing a strong capability to degrade BAs from kimchi, steady efforts and research are likely required in the future. In addition, to prove that the difference in the BA degradation activity of LAB is at least partly due to the degradable BA content of the foods (i.e., protein-poor and protein-rich foods) from which they are isolated, a survey study comparing the BA degradation activity of LAB isolated from various foods with different protein content is also required.

Meanwhile, the BA-producing ability of the five LAB strains selected in this study had previously been examined by Jin et al. [32] and Lee et al. [34]. In the previous reports, the five LAB strains displayed low (below 3 μg/mL) or no production of eight kinds of BAs, and, particularly, no production of both toxic BAs. Although one of the five LAB strains was identified as *L. brevis*, the other LAB strains not included in the five strains but identified as *L. brevis* were found to produce large quantities of BAs. Together with the toxic BA degradation results of the *L. brevis* PK08 strain described above, the results of these studies support other previous reports suggesting that BA-producing and toxic BA-degrading capabilities depend not only on the species but also on the strains. Consequently, the five LAB strains capable of degrading toxic BAs and incapable of producing both toxic BAs were isolated from kimchi varieties in this study. It is expected that BA concentrations in kimchi could be reduced by the use of the selected LAB strains as starter or protective cultures in fermentation.

### 3.2. Changes in Physicochemical and Microbiological Parameters during Fermentation of Baechu Kimchi Inoculated with BA-Degrading LAB Strains

Fermentation of *Baechu* kimchi was carried out to practically investigate the influences of the strains selected above (*L. brevis* PK08, *L. pentosus* PK05, *Leu. mesenteroides* YM20, *L. plantarum* KD15, and *L. sakei* YM21) on the physicochemical and microbial properties (Section 3.2) as well as on the BA content in kimchi (Section 3.3).

**Figure 1:**
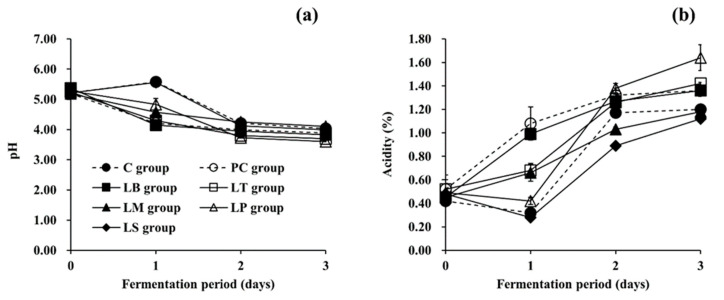
Changes in physicochemical properties of *Baechu* kimchi during fermentation. (**a**) pH, (**b**) acidity. ●: C group without an inoculum (control), ○: PC group inoculated with tyramine-producing *L. brevis* isolated from kimchi product (positive control), ■: LB group inoculated with BA-degrading *L. brevis* PK08, □: LT group inoculated with BA-degrading *L. pentosus* PK05, ▲: LM group inoculated with BA-degrading *Leu. mesenteroides* YM20, △: LP group inoculated with BA-degrading *L. plantarum* KD15, ◆: LS group inoculated with BA-degrading *L. sakei* YM21.

The changes in physicochemical properties (Figure 1) of the non-inoculated group (C group, naturally fermented control) and all inoculated groups (PC group, positive control inoculated with a tyramine-producing LAB strain; LB, LT, LM, LP, and LS groups inoculated with toxic BA-degrading LAB strains; refer to Section 2.4) were consistent with those of a previous study described by Mheen and Kwon [54], which reported on kimchi fermented under conditions similar to the current study. 

**Figure 2:**
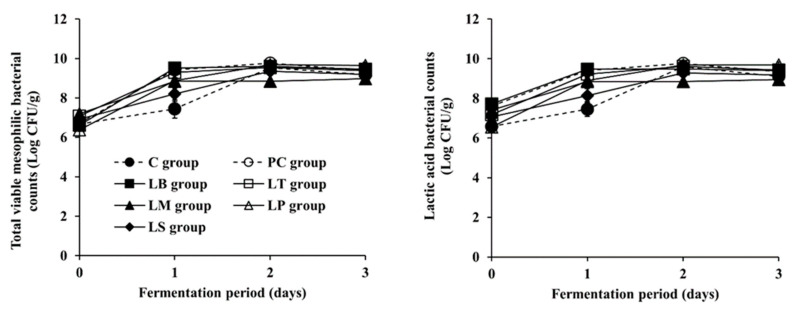
Changes in microbial properties of *Baechu* kimchi during fermentation. ●: C group without an inoculum (control), ○: PC group inoculated with tyramine-producing *L. brevis* isolated from kimchi product (positive control), ■: LB group inoculated with BA-degrading *L. brevis* PK08, □: LT group inoculated with BA-degrading *L. pentosus* PK05, ▲: LM group inoculated with BA-degrading *Leu. mesenteroides* YM20, △: LP group inoculated with BA-degrading *L. plantarum* KD15, ◆: LS group inoculated with BA-degrading *L. sakei* YM21.

The initial pH values of all groups were determined to be in the range of 5.19 ± 0.04 to 5.37 ± 0.16 (5.24 ± 0.06, mean ± standard deviation from the values of all the groups), as shown in Figure 1a. The pH of the C and LS groups increased slightly during the first day of fermentation, and thereafter decreased steadily to 4.03 ± 0.04 and 3.99 ± 0.02, respectively. This slight increase in pH is consistent with previous reports describing the fermentation of other kimchi varieties [32,34]. Meanwhile, the pH of the other groups (PC, LB, LT, LM, and LP groups) decreased gradually to the range of 3.60 ± 0.01 to 4.10 ± 0.04 throughout the fermentation period. The differences in pH variation among the groups as fermentation progressed is likely connected with the counts of total viable mesophilic bacteria, particularly LAB, as seen in Figure 2. In this regard, it is notable that the rise in microbial counts of the C and LS groups on day one was slightly slower than those of the other groups (PC, LB, LT, LM, and LP groups). Thus, it seems that slower microbial growth, compared to the other groups, caused delayed production of organic acids, which in turn, affected the pH (and acidity, as well) of both groups.

Meanwhile, as shown in Figure 1b, the initial titratable acidity of all groups was in the range of 0.42 ± 0.04 to 0.52 ± 0.12% (0.48 ± 0.04%, mean ± standard deviation from the values of all the groups). As for the C and LS groups, the titratable acidity was observed to decrease slightly on the first day of fermentation and then increased gradually to 1.20 ± 0.02% and 1.12 ± 0.02%, respectively, towards the end of the fermentation process. Contrastingly, the titratable acidity of the LP group remained statistically unchanged (but decreased slightly) on day one compared to the initial acidity and rose thereafter, while that of the PC, LB, LT, and LM groups increased throughout the whole fermentation period. On day three of fermentation, the titratable acidity of the PC, LB, LT, LM, and LP groups eventually reached a range of 1.18 ± 0.06 to 1.64 ± 0.11%. These changes in acidity throughout the fermentation period corresponded with the changes in pH for all groups. In other words, a rise in pH was seen concurrently with a decrease in acidity, and vice versa. It is also worth noting that the largest increases in acidity on day one were seen in the PC and LB groups, while the LP group displayed the largest acidity increase each day thereafter. In any case, the titratable acidity of all groups reached over 1.0% by the third day of fermentation, indicating that the *Baechu* kimchi was over-ripened. Kimchi ripeness is commonly divided into less ripened, optimally ripened, and over-ripened, based on pH and acidity. Mheen and Kwon [54] suggested that the pH and acidity of optimally ripened kimchi are in the ranges of 4.2 to 4.5 and 0.6 to 1.0%, respectively, while the acidity of over-ripened kimchi is over 1.0%. According to these criteria, the PC group had already reached the over-ripened level on day one. Meanwhile, optimal fermentation of the LB, LT, and LM groups was observed on the first day, and on day two for the LS group. Interestingly, the LP group was less ripened on day one, but found to be over-ripened on day two, without observation of a state of optimal ripening. The C group followed a similar pattern as the LP group. Thus, it is expected that kimchi fermentation can be controlled by the use of selected LAB strains with specific effects on kimchi acidity, to give a final product with a desired level of ripeness for any given length of fermentation.

The inoculum size for all inoculated groups was at the same level of 7 Log CFU/mL, but the initial counts of total viable mesophilic bacteria and LAB immediately after *Baechu* kimchi preparation were somewhat different among the groups, as shown in Figure 2. In detail, the initial total viable mesophilic bacteria and LAB counts of the C group were 6.67 ± 0.06 Log CFU/mL and 6.59 ± 0.13 Log CFU/mL, respectively. The LP group showed the lowest initial counts of total viable mesophilic bacteria and LAB, 6.38 ± 0.06 Log CFU/mL and 6.56 ± 0.09 Log CFU/mL, while those of the other inoculated groups (PC, LB, LT, LM, and LS groups) were higher than the C group by up to 0.53 Log CFU/mL and 1.13 Log CFU/mL, respectively. Therefore, considering that the inoculum size for each group was at the same level, the adaptability of LAB strains to the kimchi environment seems to differ depending on the species and/or strains, which may eventually lead to different initial counts of total viable mesophilic bacteria and LAB. In any case, the counts of all groups increased throughout the kimchi fermentation. However, on the first day of fermentation, the counts of the C and LS groups were 7.44 ± 0.46 and 8.20 ± 0.38 Log CFU/mL for total viable mesophilic bacteria, respectively, and 7.46 ± 0.37 and 8.13 ± 0.35 Log CFU/mL for LAB, respectively, while those of the other groups (PC, LB, LT, LM, and LP groups) were higher, in the ranges of 8.85 ± 0.06 to 9.51 ± 0.10 Log CFU/mL for total viable mesophilic bacteria and 8.84 ± 0.03 to 9.47 ± 0.09 Log CFU/mL for LAB. Nevertheless, the counts of total viable mesophilic bacteria and LAB of the C and LS groups increased faster than the other groups between the first and second days of fermentation. Thus, the counts of total viable mesophilic bacteria and LAB of all groups reached the ranges of 8.85 ± 0.07 to 9.76 ± 0.07 Log CFU/mL and 8.84 ± 0.05 to 9.76 ± 0.06 Log CFU/mL, respectively, by day two of fermentation, and remained constant thereafter. These results support the assumption that slower microbial growth observed in both the C and LS groups might result in the highest pH and the lowest acidity, as aforementioned. In addition, in all groups, the counts of the total viable mesophilic bacteria were observed to be similar to those of the LAB during the fermentation period, which indicates that the LAB were the predominant microorganisms in the *Baechu* kimchi. Altogether, the results of physicochemical and microbial measurements indicate that all *Baechu* kimchi groups, regardless of inoculum, were properly fermented.

### 3.3. Changes in BA Content during Fermentation of Baechu Kimchi Inoculated with BA-Degrading LAB Strains

**Figure 3:**
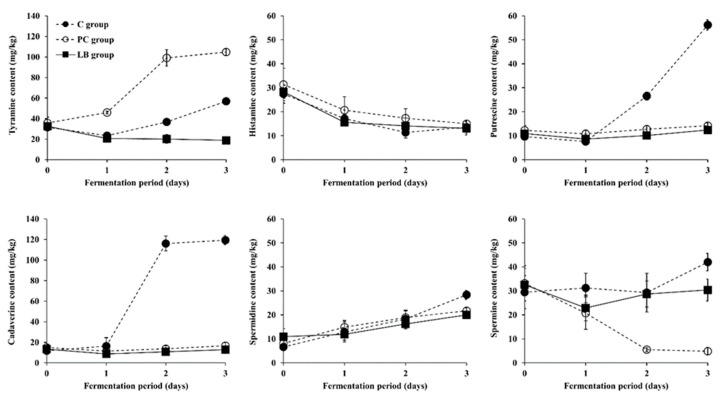
Changes in BA content in *Baechu* kimchi inoculated with a LAB strain leading to significant tyramine reduction during fermentation. ●: C group without an inoculum (control), ○: PC group inoculated with tyramine-producing *L. brevis* isolated from kimchi product (positive control), ■: LB group inoculated with BA-degrading *L. brevis* PK08.

**Figure 4:**
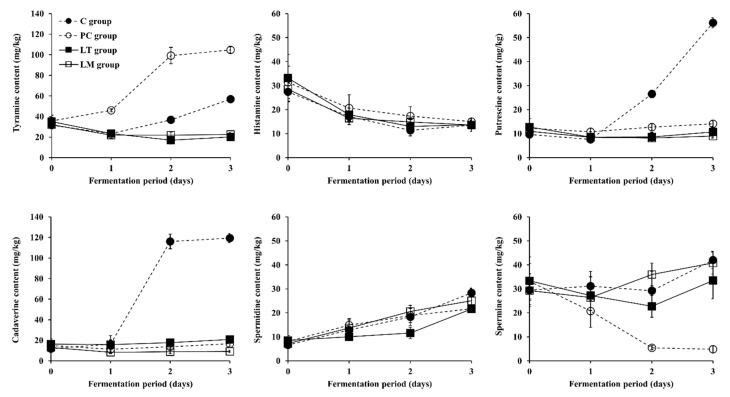
Changes in BA content in *Baechu* kimchi inoculated with LAB strains leading to moderate tyramine reduction during fermentation. ●: C group without an inoculum (control), ○: PC group inoculated with tyramine-producing *L. brevis* isolated from kimchi product (positive control), ■: LT group inoculated with BA-degrading *L. pentosus* PK05, □: LM group inoculated with BA-degrading *Leu. mesenteroides* YM20.

Through the *Baechu* kimchi fermentation experiments described in Section 3.2, it was also evaluated whether the selected LAB strains with the capability of toxic BA degradation would practically reduce the content of BAs (particularly tyramine) in *Baechu* kimchi. Changes in the content of six kinds of BAs, including tyramine, histamine, putrescine, cadaverine, spermidine, and spermine, during *Baechu* kimchi fermentation, are presented in Figure 3 and Figure 4, and Figure S1 and Table S3. Tryptamine and β-phenylethylamine were also determined but are not described hereafter, as the tryptamine content in all groups was lower than 10 mg/kg throughout the fermentation period, and β-phenylethylamine was not detected in all groups during fermentation (data not shown).

As shown in Figure 3, the tyramine, putrescine, cadaverine, spermidine, and spermine content of the C group (naturally fermented control without any inoculum) consistently increased between days one and three of fermentation, while the histamine content steadily deceased throughout the fermentation period. On day three of fermentation, this increased content of the five BAs of the C group was the highest among all the experimental *Baechu* kimchi groups (except for tyramine content in the PC and LS groups described below; also refer to Figure 3 and Figure S1, respectively), but the decrease in histamine content was similar to the inoculated groups. These results were likely associated with the BA-producing and BA-degrading activities of some indigenous LAB strains, and are in accordance with observations from previous reports [32,34]. As suggested by Dierick et al. [55] and reviewed by Park et al. [30] and Barbieri et al. [56], the accumulation of BAs in fermented food products results from the decarboxylation of free amino acids by the amino acid-decarboxylase activity of some LAB species present in the food. Furthermore, other previous studies have reported that LAB species, including *L. plantarum*, *L. brevis*, *L. curvatus*, *Lentilactobacillus hilgardii* (formerly *Lactobacilus hilgardii*), *L. casei*, and *Leu. mesenteroides*, were likely responsible for BA production in fermented foods such as wine, cheese, cider, beer, and kimchi, and had genes encoding amino acid decarboxylases [50,57,58,59,60]. Therefore, BA accumulation seems to be an unavoidable phenomenon in naturally fermented kimchi. 

Meanwhile, the tyramine, putrescine, cadaverine, and spermidine content of the PC group inoculated with the tyramine-producing *L. brevis* PK11 strain steadily increased during the fermentation period, while histamine and spermine content gradually decreased (Figure 3). Among the four BAs that increased, the tyramine content of the PC group (104.74 ± 3.04 mg/kg) on day three of fermentation was at least two times higher than that of the other groups (for instance, 56.88 ± 1.25 mg/kg for C group) and was over the harmful level (100 mg/kg) of tyramine [8]. The observation was most likely due to the *L. brevis* PK11 strain being used as a tyramine-producing reference strain (but incapable of producing the other BAs). The content of putrescine, cadaverine, and spermidine was lower in the PC group than in the C group on day three. *L. brevis* PK11 is hence believed to be implicated in this difference, as the polyamine-producing strains present in the naturally fermented *Baechu* kimchi (C group) might have been restricted by this inoculated strain in the PC group, and thus not be able to produce the aforementioned BAs. In the meantime, the decrease in histamine content of the PC group throughout the fermentation period was consistent with that of the C group. The spermine content of the PC group on days two and three, however, was the lowest among the groups. On the aspect of correlation between the changes in tyramine and spermine content of the PC group, interestingly, as the spermine content decreased during fermentation, the tyramine content increased (R^2^ = 0.93). A similar correlation has also been observed in previous studies [32,34]. Thus, the microbial production of tyramine seems to be negatively related to spermine content in *Baechu* kimchi. Further research is required to elucidate this assumption. In regard to the BA content of kimchi, only a small number of studies have been conducted [30]. Nevertheless, some reports have described that BA content in kimchi is within the acceptable ranges for human health [29,61], however, kimchi with BA levels over the acceptable ranges has also been reported [31,33,35]. In addition, the current commercial production of kimchi depends largely on natural fermentation, because the application of starter cultures may affect the flavor and quality of kimchi products and there is no scientific guideline for the kimchi manufacturing process, such as the salting of napa cabbage and the ripening of the kimchi product [62,63]. Besides, considerable tyramine content was measured in the PC group inoculated with tyramine-producing *L. brevis* PK11, as mentioned above. Consequently, the presence of LAB strains possessing the ability to produce BAs, such as *L. brevis* PK11, isolated from a kimchi variety in this study, in kimchi products may lead to the accumulation of BAs as kimchi fermentation progresses, which may potentiate food safety risks associated with BAs (particularly tyramine). For this reason, efforts to find or develop BA-degrading LAB strains with weak or no BA-producing capability to be used as starter or protective cultures for kimchi fermentation are suggested for further research to reduce the content of BAs in kimchi products.

In contrast to the C and PC groups, as shown in Figure 3, the content of both tyramine and histamine of the LB group gradually decreased during fermentation. The cadaverine and spermine content also slightly, but statistically insignificantly, decreased, while the putrescine and spermidine content slightly, but statistically significantly, increased throughout the fermentation period. Overall, the content of the six BAs at the end of the fermentation period was remarkably lower in the LB group than in both the C group and the PC group (except for spermine content in the PC group). Particularly, the tyramine content was reduced by 66.65% and 81.89% compared to the C and PC groups, respectively. As discussed in Section 3.1, the five selected LAB strains had the potential to be used as toxic BA-degrading starter or protective cultures for the reduction in BA content in kimchi fermentation. Indeed, the toxic BA degradation capability of the *L. brevis* PK08 strain was validated not only in the in vitro BA degradation test (Section 3.1) but also in the *Baechu* kimchi fermentation experiment (Section 3.3). As for the change in tyramine content, a considerable decrease was observed in the LB group compared to that of the C and PC groups. Possible explanations for this result may include: (i) antimicrobial activity against BA-producing microorganisms, (ii) inactive BA production, and/or (iii) BA degradation activity of *L. brevis* PK08 strain. Regarding the first assumption, although the antimicrobial activity of all the selected LAB strains, including *L. brevis* PK08 strain, was tested in this study, none of the LAB strains were found to have antimicrobial activity against BA-producing LAB strains (data not shown). Thus, it seems that the other reasons are more probable. As for the change in histamine content, however, a gradual decrease during fermentation was observed in all groups, including the C group that was fermented naturally. Also, as presented in Table 1, the histamine degradation of the *L. brevis* PK08 strain (and the other selected LAB strains) was lower than the tyramine degradation in both buffer and culture media. Therefore, it is unclear whether the decrease in histamine content of the LB group is attributed to degradation activity of the inoculated strain, *L. brevis* PK08. This is also the case for the other inoculated groups. Although further research on the kinetics and mechanism of histamine degradation during kimchi fermentation is suggested, it is evident that the *L. brevis* PK08 strain isolated from a kimchi variety somehow showed effective toxic BA degradation activity (particularly tyramine) during *Baechu* kimchi fermentation. It is also notable that the accumulation of all six BAs, including polyamines as well as toxic BAs, was suppressed in the LB group, compared to the C group that was fermented naturally, as described above. Similar to the PC group, it seems that the polyamine-producing strains present in the naturally fermented *Baechu* kimchi were somehow restricted by the *L. brevis* PK08 strain in the LB group. Altogether, the present study suggests that LAB strains capable of degrading toxic BAs, such as *L. brevis* PK08, may be worthwhile to use for the prevention of food safety issues associated not only with toxic BAs but also with polyamines in kimchi.

As shown in Figure 4, the changes in BA content of the LT and LM groups, inoculated with the *L. pentosus* PK05 strain or the *Leu. mesenteroides* YM20 strain, respectively, were determined to be similar to the patterns observed in the LB group. However, at the end of fermentation, the tyramine, histamine, spermidine, and spermine content of both the LT and LM groups were slightly higher than those of the LB group, while the putrescine content was slightly lower. Compared to the LB group, the cadaverine content was slightly higher in the LT group but slightly lower in the LM group. Nevertheless, the differences in each of the six BA contents were statistically insignificant among the three groups. Meanwhile, the LP and LS groups inoculated with the *L. plantarum* KD15 strain or the *L. sakei* YM21 strain, respectively, were also tested, but are not described hereafter because the groups did not show a strong effect on BA degradation activity in *Baechu* kimchi compared to the other groups (LB, LT, and LM groups; Figure S1). Based on the in vitro toxic BA degradation test (Table 1), it was expected that *L. sakei* YM21 would have moderate degradation activity of both tyramine and histamine in the *Baechu* kimchi fermentation experiment, and *L. plantarum* KD15 and *Leu. mesenteroides* YM20, which showed the highest and second-highest tyramine degradation activity in vitro, would also perform highly in fermentation experiments. Contrary to this expectation, *L. brevis* PK08 showed the most remarkable toxic BA degradation (particularly tyramine), while *L. sakei* YM21, *L. plantarum* KD15, and *Leu. mesenteroides* YM20 underperformed. Such results indicate that the use of only in vitro testing has limitations in selecting the optimal LAB strains capable of degrading BAs in practical fermentation. Therefore, as in this study, the best strategy for choosing an optimal strain for fermentation would be to shortlist a few high performing strains from the in vitro tests and apply them to the fermentation experiments, then select the optimal strain. In addition, since the level of *Baechu* kimchi ripeness at a given time differs due to how inocula of different LAB species affect acidity (as described in Section 3.2), it can be expected that the control of kimchi fermentation by the use of selected LAB strains as starter and/or protective cultures can not only reduce the BA content of the resulting product, but a favorable level of ripeness at a desired time can also be achieved.

### 3.4. Contribution of Multicopper Oxidase Gene in L. brevis Strains to BA Degradation

**Figure 5:**
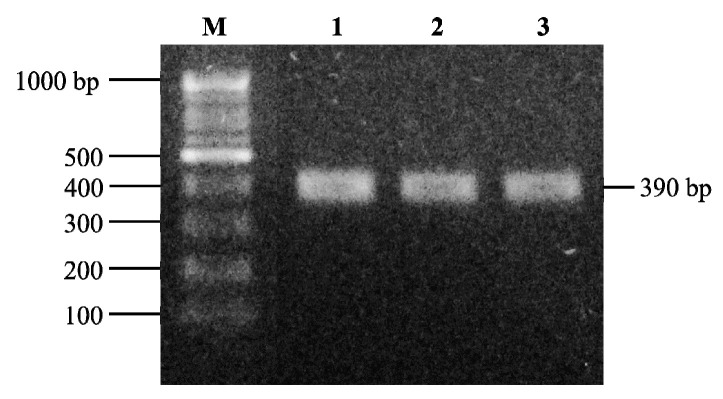
Electrophoresis of PCR products amplified for a gene encoding MCO (390 bp) in *L. brevis*. Lanes M: 1000 bp DNA ladder, 1: *L. brevis* JCM 1170, 2: *L. brevis* PK08, 3: *L. brevis* PK11. The MCO gene was not detected in *L. helveticus* KCCM 40989, *L. plantarum* KCTC 3108, *L. sakei* KCCM 43213, *L. casei* KCCM 12452, *L. paracasei* KCTC 3510, *L. fermentum* KCTC 3112, *L. buchneri* KCTC 5064, *Leu. mesenteroides* KCTC 3505, and *Leu. citreum* KCCM 12030.

Previous studies have reported that LAB strains such as *L. curvatus*, *L. plantarum*, and *L. paracasei* could degrade BAs, including tyramine, histamine, and putrescine, in fermented foods such as fermented meat, wine, and cheese [38,41,42]. These studies have also described that the multicopper oxidase (MCO) genes which encode enzymes responsible for BA degradation are present in the LAB strains. The activity of such BA-degrading enzymes including MCOs and amine oxidases may provide substances that can be used as sources of energy and growth [64]. Other amine oxidase genes in LAB, such as monoamine oxidase, polyamine oxidase, and copper-containing amine oxidases, have rarely been registered in NCBI. As shown in Figure 5, therefore, it was examined whether the strain with the highest BA-degrading activity from the *Baechu* kimchi fermentation experiment, *L. brevis* PK08 (and *L. brevis* PK11 and *L. brevis* JCM 1170), carries the gene coding for a BA-degrading MCO. Using the primer set described in Section 2.7, the MCO gene was detected in all three *L. brevis* strains, but not detected in the other nine LAB species examined. The sequences of the detected genes showed high levels of identity (higher than 99%) with *L. brevis* MCO gene (accession number CP031208.1). Thus, it is clear that MCO is responsible for BA degradation (particularly tyramine) of the *L. brevis* strains during *Baechu* kimchi fermentation in this study. It is noteworthy that glyceraldehyde-3-phosphate dehydrogenase (GAPDH) has been identified in *L. plantarum* as an enzyme capable of degrading histamine [65]. Together with the fact that GAPDH is produced by *L. brevis* (accession number QCZ55221), both MCO and GAPDH may have a role in BA degradation in kimchi. However, there is still a lack of available information on amine oxidase genes in LAB species, with the exception of MCO and GAPDH, as aforementioned. Apart from this, since the MCO genes are found in the genomes of *L. pentosus*, *Leu. mesenteroides*, *L. plantarum*, and *L. sakei* that are registered in NCBI, it is likely that in a future study, a universal-primer or multi-primer set could be developed for the rapid and simultaneous detection of the MCO genes in the LAB species.

Although *L. brevis* PK11 carried the MCO gene and showed tyramine-degrading activity in the toxic BA degradation test (data not shown), used as the positive control (PC group) in the *Baechu* kimchi fermentation experiment, tyramine content increased considerably. This indicates that when selecting a starter or protective culture that reduces the BA content of a fermented food product, in addition to BA degrading activity, BA production must also be considered.

## 4. Conclusions

In the present study, the highest BA levels (except for tyramine) among all the experimental *Baechu* kimchi groups were seen in the C group, fermented naturally without a LAB inoculum, which seems to be associated with the BA-producing activity of those indigenous LAB species and/or strains present in the kimchi that are commonly responsible for BA production in lactic acid fermented foods, as reported in other previous studies. Based on observations in previous and present studies, BA accumulation seems to be inevitable in naturally fermented kimchi. In addition, the tyramine content of the PC group inoculated with tyramine-producing *L. brevis* PK11, isolated from a kimchi variety, was the highest among all the groups, exceeding the harmful level (100 mg/kg). This means that food safety risks associated with BAs (particularly tyramine) in kimchi will be greater when prolific tyramine-producing LAB strains such as *L. brevis* PK11 are the dominant indigenous strains. Therefore, starter or protective cultures should be developed to reduce the BA content in kimchi. Meanwhile, certain LAB, such as *L. brevis* PK11, with BA degradation capability are also able to largely produce BAs, leading to increased BA content in fermented kimchi. This suggests that when selecting possible LAB starter and/or protective cultures, both BA degradation and BA production must be considered in order to ensure the reduction in BA content in kimchi products. 

Considering these selecting criteria, the present study found that the five LAB strains isolated from different kimchi varieties that were capable of degrading both toxic BAs (except for one strain) and incapable of producing both toxic BAs in vitro also had the capability to degrade the toxic BAs in *Baechu* kimchi fermentation experiments. Through the *Baechu* kimchi fermentation, it was observed that *L. brevis* PK08 considerably reduced all six BAs, including polyamines as well as toxic BAs. Thus, the highest tyramine reduction compared to both the C group and PC group was seen in the LB group inoculated with *L. brevis* PK08, relative to the other inoculated groups, at rates of 66.65% and 81.89%, respectively, while relatively high histamine, putrescine, cadaverine, spermidine, and spermine reductions were also seen. In addition, a gene that codes a BA-degrading enzyme, MCO, was detected in *L. brevis* PK08, which means that MCO is (at least in part) responsible for the BA reduction. The other LAB strains showed similar or less BA reduction in *Baechu* kimchi than *L. brevis* PK08. Meanwhile, the optimal fermentation of each group was observed at different fermentation periods depending on the species and/or strains, because their distinct growth affected the pH and acidity of *Baechu* kimchi differently, thus determining the level of ripeness. Taken together, this study suggests that the LAB strains are valuable as starter or protective cultures in kimchi fermentation to reduce BA content and prevent BA-related food safety issues, and through the control of kimchi fermentation by the use of selected LAB strains, favorable levels of ripeness can also be achieved.

## Figures and Tables

**Table 1:** Degradation rates of histamine and tyramine in buffer and media by selected LAB strains originated from kimchi varieties.

Kimchi Isolate No. ^1^	Buffer		Media		Identification
	Degradation (%)		Degradation (%)		
	HIS ^2^	TYR ^2^	HIS	TYR	
PK08	3.65–8.58 ^3^ (6.12 ± 3.49) ^a^	3.50–11.54 (7.52 ± 5.69) ^a^	5.26–8.56 (6.91 ± 2.33) ^a^	1.82–11.68 (6.75 ± 6.97) ^a^	*Levilactobacillus brevis*
PK05	7.47–10.81 (9.14 ± 2.36) ^a^	5.25–7.43 (6.34 ± 1.54) ^a^	1.17–1.59 (1.38 ± 0.30) ^b^	2.97–13.13 (8.05 ± 7.18) ^a^	*Lactiplantibacillus pentosus*
YM20	ND–1.39 (0.70 ± 0.98) ^b^	3.25–11.90 (7.58 ± 6.12) ^a^	4.09–4.28 (4.19 ± 0.13) ^a^	2.11–14.36 (8.24 ± 8.66) ^a^	*Leuconostoc mesenteroides*
YM21	0.21–10.54 (5.38 ± 7.30) ^a^	3.47–11.41 (7.44 ± 5.61) ^a^	0.58–7.96 (4.27 ± 5.22) ^a^	2.39–12.11 (7.25 ± 6.87) ^a^	*Latilactobacillus sakei*
KD15	3.64–5.67 (4.66 ± 1.44) ^a^	4.03–14.97 (9.50 ± 7.74) ^a^	ND–4.34 (2.17 ± 3.07) ^a^	2.39–14.88 (8.64 ± 8.83) ^a^	*Lactiplantibacillus plantarum*

^1^ PK: *Pa* kimchi (kimchi made mainly of green onion), YM: *Yeolmu* kimchi (kimchi made mainly of young radish), KD: *Kkakdugi* (kimchi made mainly of diced radish). ^2^ HIS: histamine, TYR: tyramine. ^3^ Values represent the minimum and maximum (mean ± standard deviation) as determined by duplicate experiments. Mean values in the same row of the same BA followed by a different letter (a–b) are significantly different (*p* < 0.05), ND: no degradation detected.

## Supplementary Materials

The following are available online at https://www.mdpi.com/article/10.3390/microorganisms9122570/s1, Figure S1: Changes in BA content in *Baechu* kimchi inoculated with other selected LAB strains during fermentation, Table S1: Histamine and tyramine degradation by LAB strains previously isolated from kimchi varieties, Table S2: Histamine and tyramine degradation by LAB strains isolated from kimchi varieties in this study, Table S3: Changes in BA content during fermentation of *Baechu* kimchi inoculated with different LAB strains capable of degrading toxic BAs.
