# Supplementary material for: Reduction in Biogenic Amine Content in Baechu (Napa Cabbage) Kimchi by Biogenic Amine-Degrading Lactic Acid Bacteria
Source: Microorganisms. 2021 Dec 13;9(12):2570. doi: 10.3390/microorganisms9122570 (PMC8704687; doi:10.3390/microorganisms9122570)
Supplement: Supplementary file 1 [file microorganisms-09-02570-s001.zip › Figure S1.pdf]

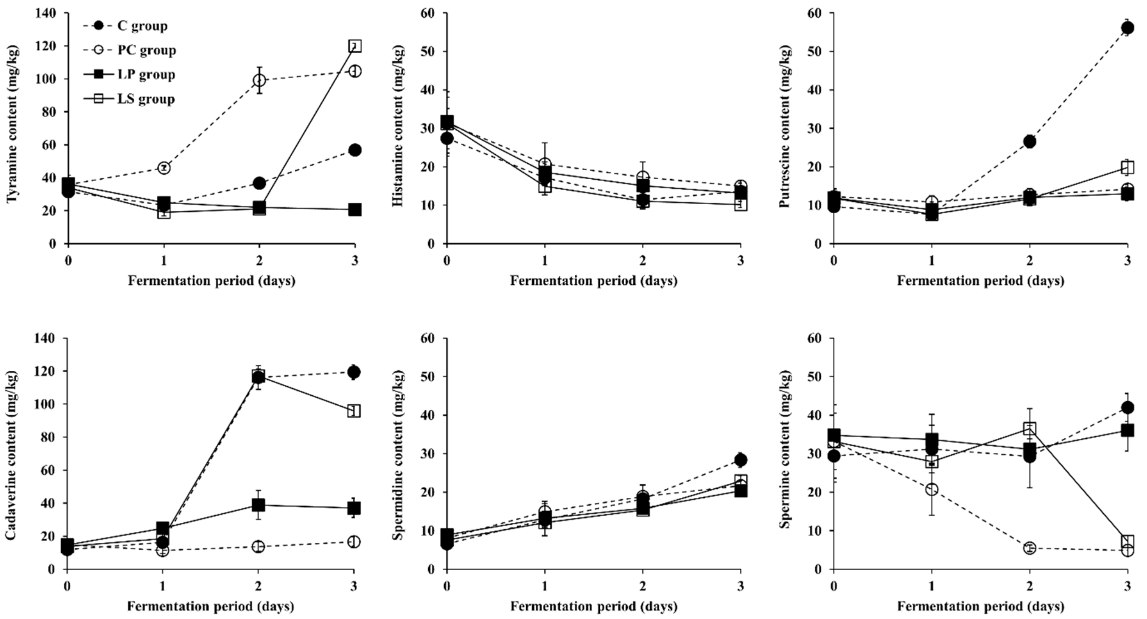

Figure S1. Changes in BA content in *Baechu* kimchi inoculated with other selected LAB strains during fermentation. ●: C group without an inoculum (control), ○: PC group inoculated with tyramine-producing *L. brevis* isolated from kimchi product (positive control), ■: LP group inoculated with BA-degrading *L. plantarum* KD15, □: LS group inoculated with BA-degrading *L. sakei* YM21. Error bars indicate the standard deviations determined from duplicate experiments.
