# Supplementary material for: Reduction in Biogenic Amine Content in Baechu (Napa Cabbage) Kimchi by Biogenic Amine-Degrading Lactic Acid Bacteria
Source: Microorganisms. 2021 Dec 13;9(12):2570. doi: 10.3390/microorganisms9122570 (PMC8704687; doi:10.3390/microorganisms9122570)
Supplement: Supplementary file 1 [file microorganisms-09-02570-s001.zip › Tables S1-S3.pdf]

Table S1. Histamine and tyramine degradation by LAB strains previously isolated from kimchi varieties.

Kimchi Samples		Number of Isolates	Number of BA-Degrading LAB Isolates (ND / ≤ 10% / > 10%) [4]	
Kimchi Varieties [1]	N [2]	N_{iso} (N_{deg}) [3]	HIS [5]	TYR
BC	5	97 (0)	46/51/0	50/47/0
KD	5	88 (4)	34/51/3	20/66/2
CG	5	105 (2)	27/77/1	23/80/2
YM	5	106 (5)	39/64/3	40/61/5
GK	5	83 (8)	11/64/8	8/73/2
PK	5	53 (7)	3/44/6	6/42/5

[1] BC: *Baechu* kimchi (kimchi made mainly of napa cabbage), KD: *Kkakdugi* (kimchi made mainly of diced radish), CG: *Chonggak* kimchi (kimchi made mainly of ponytail radish), YM: *Yeolmu* kimchi (kimchi made mainly of young radish), GK: *Gat* kimchi (kimchi made mainly of mustard leaf), PK: *Pa* kimchi (kimchi made mainly of green onion).

[2] N: the number of samples examined.

[3] N_{iso}: the number of isolated LAB strains from each kimchi sample, N_{deg}: the number of LAB strains showing a degradation rate of histamine or tyramine over 10%.

[4] The number of toxic BA-degrading LAB strains classified by degradation range: ND (not detected), ≤ 10%, > 10% for each BA. The BA-degrading ability of LAB strains was tested in buffer.

[5] HIS: histamine, TYR: tyramine.

Table S2. Histamine and tyramine degradation by LAB strains isolated from kimchi varieties in this study.

Kimchi Samples		Number of Isolates	Number of BA-Degrading LAB Isolates (ND / ≤ 10% / > 10%) [4]	
Kimchi Varieties [1]	N [2]	N_{iso} (N_{deg}) [3]	HIS [5]	TYR
BC	12	707 (45)	188/264/15	157/303/43
KD	1	150 (3)	22/52/1	34/39/2
CG	1	59 (1)	20/5/0	14/19/1

[1] BC: *Baechu* kimchi (kimchi made mainly of napa cabbage), KD: *Kkakdugi* (kimchi made mainly of diced radish), CG: *Chonggak* kimchi (kimchi made mainly of ponytail radish).

[2] N: the number of samples examined.

[3] N_{iso}: the number of isolated LAB strains from each kimchi sample, N_{deg}: the number of LAB strains showing a degradation rate of histamine or tyramine over 10%.

[4] The number of BA-degrading isolates in each degradation range was indicated as follow: ND (not detected), ≤ 10%, > 10% The number of toxic BA-degrading LAB strains classified by degradation range: ND (not detected), ≤ 10%, > 10% for each BA. The BA-degrading ability of LAB strains was tested in buffer or culture media.

[5] HIS: histamine, TYR: tyramine.

Table S3. Changes in BA content during fermentation of *Baechu* kimchi inoculated with different LAB strains capable of degrading toxic BAs.

Kimchi Groups [1]	Day [2]	BA Content (mg/kg) [3]					
		TYR	HIS	PUT	CAD	SPD	SPM
C group	0	31.54 ± 1.68 $^{A, b}$	27.40 ± 3.84 $^{A, a}$	9.66 ± 0.59 $^{B, b}$	11.91 ± 2.05 $^{B, b}$	6.62 ± 0.53 $^{B, b}$	29.46 ± 6.87 $^{A, a}$
	1	23.35 ± 2.68 $^{BC, b}$	17.18 ± 1.69 $^{AB, b}$	7.60 ± 0.61 $^{B, b}$	16.36 ± 8.18 $^{ABC, b}$	12.87 ± 4.18 $^{AB, b}$	31.20 ± 6.16 $^{AB, a}$
	2	36.80 ± 1.43 $^{B, ab}$	11.39 ± 2.32 $^{BC, c}$	26.56 ± 1.65 $^{A, b}$	116.11 ± 7.20 $^{A, a}$	18.31 ± 3.69 $^{AB, ab}$	29.26 ± 8.06 $^{ABC, a}$
	3	56.88 ± 1.25 $^{C, a}$	13.57 ± 2.67 $^{A, bc}$	56.20 ± 2.15 $^{A, a}$	119.42 ± 4.40 $^{A, a}$	28.37 ± 1.88 $^{A, a}$	42.01 ± 3.63 $^{A, a}$
PC group	0	35.83 ± 5.69 $^{A, c}$	31.37 ± 6.75 $^{A, a}$	12.25 ± 2.01 $^{AB, ab}$	14.89 ± 3.04 $^{AB, ab}$	8.09 ± 0.86 $^{B, c}$	33.19 ± 7.31 $^{A, a}$
	1	45.95 ± 1.36 $^{A, b}$	20.69 ± 5.53 $^{A, b}$	10.81 ± 1.63 $^{A, b}$	11.40 ± 1.96 $^{BC, b}$	14.91 ± 2.69 $^{A, b}$	20.75 ± 6.74 $^{C, b}$
	2	99.18 ± 7.99 $^{A, a}$	17.30 ± 3.94 $^{A, b}$	12.72 ± 1.55 $^{B, ab}$	13.69 ± 3.03 $^{B, ab}$	18.89 ± 2.86 $^{AB, a}$	5.48 ± 0.84 $^{D, c}$
	3	104.74 ± 3.04 $^{B, a}$	15.02 ± 0.68 $^{A, b}$	14.16 ± 1.24 $^{B, a}$	16.72 ± 2.86 $^{B, a}$	21.72 ± 1.23 $^{A, a}$	4.88 ± 1.49 $^{B, c}$
LB group	0	32.64 ± 0.93 $^{A, a}$	28.34 ± 2.74 $^{A, a}$	10.93 ± 0.58 $^{AB, ab}$	13.36 ± 1.56 $^{AB, a}$	10.91 ± 3.38 $^{A, c}$	32.58 ± 6.74 $^{A, a}$
	1	20.72 ± 1.25 $^{CD, b}$	15.58 ± 1.21 $^{B, b}$	8.65 ± 1.61 $^{B, c}$	8.69 ± 0.26 $^{C, b}$	11.94 ± 2.52 $^{AB, c}$	22.87 ± 5.34 $^{BC, b}$
	2	20.14 ± 4.23 $^{C, b}$	14.11 ± 3.29 $^{ABC, b}$	10.08 ± 0.83 $^{B, bc}$	10.78 ± 1.43 $^{B, ab}$	16.25 ± 2.05 $^{B, b}$	28.71 ± 5.45 $^{BC, ab}$
	3	18.97 ± 3.14 $^{D, b}$	13.06 ± 2.78 $^{A, b}$	12.42 ± 0.63 $^{B, a}$	12.83 ± 2.92 $^{B, a}$	20.01 ± 1.00 $^{A, a}$	30.40 ± 4.57 $^{A, ab}$
LT group	0	35.03 ± 6.09 $^{A, a}$	33.18 ± 9.88 $^{A, a}$	12.78 ± 3.49 $^{A, a}$	16.40 ± 5.01 $^{A, a}$	8.54 ± 2.06 $^{AB, c}$	33.38 ± 9.95 $^{A, a}$
	1	23.41 ± 3.30 $^{BC, b}$	17.94 ± 2.62 $^{AB, b}$	8.57 ± 0.81 $^{B, b}$	15.84 ± 4.60 $^{ABC, a}$	10.09 ± 1.37 $^{B, bc}$	27.19 ± 7.84 $^{ABC, a}$
	2	17.06 ± 3.83 $^{C, b}$	13.05 ± 3.10 $^{BC, b}$	8.60 ± 1.97 $^{B, b}$	17.74 ± 2.42 $^{B, a}$	11.64 ± 2.38 $^{C, b}$	22.73 ± 4.59 $^{C, a}$
	3	20.12 ± 2.46 $^{D, b}$	13.72 ± 1.84 $^{A, b}$	10.69 ± 0.46 $^{B, ab}$	20.90 ± 0.66 $^{B, a}$	21.64 ± 1.52 $^{A, a}$	33.44 ± 7.61 $^{A, a}$
LM group	0	32.13 ± 1.58 $^{A, a}$	28.52 ± 2.53 $^{A, a}$	10.99 ± 0.85 $^{AB, a}$	13.14 ± 1.20 $^{AB, a}$	7.21 ± 0.31 $^{B, d}$	29.33 ± 4.04 $^{A, bc}$
	1	21.95 ± 3.42 $^{BCD, b}$	16.45 ± 2.72 $^{AB, b}$	8.49 ± 0.71 $^{B, b}$	8.43 ± 1.21 $^{C, b}$	13.69 ± 1.60 $^{A, c}$	26.44 ± 5.37 $^{ABC, c}$
	2	21.85 ± 3.03 $^{C, b}$	14.89 ± 1.97 $^{ABC, b}$	8.19 ± 0.48 $^{B, b}$	8.86 ± 1.89 $^{B, b}$	20.63 ± 2.53 $^{A, b}$	36.03 ± 4.70 $^{A, ab}$
	3	22.65 ± 1.58 $^{D, b}$	13.63 ± 1.53 $^{A, b}$	8.97 ± 0.79 $^{B, b}$	9.27 ± 1.20 $^{B, b}$	25.10 ± 2.25 $^{A, a}$	40.80 ± 4.56 $^{A, a}$
LP group	0	36.20 ± 1.56 $^{A, a}$	31.73 ± 3.42 $^{A, a}$	11.84 ± 1.56 $^{AB, a}$	14.73 ± 1.35 $^{AB, c}$	8.94 ± 1.61 $^{AB, d}$	34.78 ± 5.82 $^{A, a}$
	1	24.87 ± 1.71 $^{B, b}$	18.59 ± 2.66 $^{AB, b}$	8.84 ± 0.96 $^{B, b}$	24.74 ± 1.27 $^{A, bc}$	13.21 ± 1.89 $^{AB, c}$	33.63 ± 6.61 $^{A, a}$
	2	21.98 ± 1.70 $^{C, bc}$	15.06 ± 1.45 $^{BC, bc}$	11.99 ± 2.22 $^{B, a}$	38.88 ± 8.73 $^{B, a}$	15.84 ± 0.88 $^{B, b}$	31.13 ± 2.69 $^{AB, a}$
	3	20.79 ± 2.54 $^{D, c}$	13.11 ± 1.49 $^{A, c}$	13.00 ± 1.79 $^{B, a}$	37.15 ± 5.81 $^{B, ab}$	20.38 ± 0.69 $^{A, a}$	36.05 ± 5.39 $^{B, a}$
LS group	0	34.00 ± 5.20 $^{A, b}$	31.18 ± 8.37 $^{A, a}$	11.83 ± 2.50 $^{AB, b}$	13.80 ± 3.16 $^{AB, b}$	7.54 ± 0.99 $^{B, d}$	33.16 ± 9.50 $^{A, a}$
	1	18.98 ± 2.23 $^{D, c}$	14.92 ± 2.23 $^{B, b}$	7.64 ± 0.71 $^{B, c}$	18.48 ± 3.09 $^{AB, b}$	12.08 ± 1.20 $^{AB, c}$	27.95 ± 5.85 $^{ABC, a}$
	2	21.12 ± 2.31 $^{C, c}$	11.02 ± 1.58 $^{C, b}$	11.62 ± 1.24 $^{B, b}$	117.13 ± 4.21 $^{A, a}$	15.38 ± 1.22 $^{B, b}$	36.51 ± 5.21 $^{A, a}$
	3	119.99 ± 1.49 $^{A, a}$	10.19 ± 0.79 $^{B, b}$	19.81 ± 2.08 $^{B, a}$	95.89 ± 2.82 $^{A, a}$	22.90 ± 1.43 $^{A, a}$	7.20 ± 1.65 $^{A, b}$

Values are presented as mean ± standard deviation calculated from duplicated experiments. Values in the same column of different groups sampled on the same day followed by a different uppercase letter (A–D) are significantly different ($p < 0.05$). Values in the same column of the same group sampled on different days followed by a different lowercase letter (a–d) are significantly different ($p < 0.05$).

[1] C group: naturally fermented control, PC group: positive control inoculated with tyramine-producing *L. brevis* PK11, LB group: BA-degrading *L. brevis* PK08, LT group: BA-degrading *L. pentosus* PK05, LM group: BA-degrading *Leu. mesenteroides* YM20, LP group: BA-degrading *L. plantarum* KD15, LS group: BA-degrading *L. sakei* YM21.

[2] Fermentation period.

[3] TYR: tyramine, HIS: histamine, PUT: putrescine, CAD: cadaverine, SPD: spermidine, SPM: spermine.
